# Clinical features and the degree of cerebrovascular stenosis in different types and subtypes of cerebral watershed infarction

**DOI:** 10.1186/s12883-017-0947-6

**Published:** 2017-08-29

**Authors:** Yue Li, Man Li, Xiaoyu Zhang, Shuna Yang, Huimin Fan, Wei Qin, Lei Yang, Junliang Yuan, Wenli Hu

**Affiliations:** 1 Department of Neurology, Beijing Chao-Yang Hospital, Capital Medical University, No. 8 South Gongti Road, Chaoyang district, Beijing, 100020 China; 2 Department of Radiology, Beijing Chao-Yang Hospital, Capital Medical University, Beijing, China; 3 Department of Neurology, Qianfoshan Hospital, Shandong University, Jinan, China

**Keywords:** Cerebral watershed infarction, cerebrovascular stenosis, hemodynamic impairment, clinical features, prognosis

## Abstract

**Background:**

Whether there are differences in pathogenesis among different types and subtypes of cerebral watershed infarction (WSI) is controversial since they have been combined into a single group in most previous studies.

**Methods:**

We prospectively identified 340 supratentorial WSI patients at Beijing Chao-Yang Hospital, Capital Medical University, China and classified them based on diffusion-weighted imaging(DWI) templates. Baseline characteristics, clinical courses and neuroradiological features were compared among patients with different types and subtypes of WSI.

**Results:**

We identified 92 patients with cortical watershed infarction (CWI), 112 with internal watershed infarction (IWI) and 136 with mixed-type infarction. Compared with CWI patients, more IWI patients had critical stenosis of internal carotid artery (ICA) (*P* < 0.001). For the CWI group, patients with anterior watershed infarction (AWI) were more prone to critical ICA stenosis than those with posterior watershed infarction (PWI) (*P* = 0.011). For the IWI group, critical ICA stenosis was more prevalent in patients with partial IWI (P-IWI) than in those with confluent IWI (C-IWI) (*P* = 0.026). IWI patients were more frequently found to have clinical deterioration during the first 7 days of hospitalization and a poor prognosis at the 90th day than in CWI patients (*P* = 0.003 and *P* = 0.014, respectively).

**Conclusions:**

IWI, especially the P-IWI subtype, is associated with hemodynamic impairment (HDI), whereas CWI has a weaker correlation with ICA steno-occlusion. Furthermore, IWI patients are more prone to poor prognosis.

**Electronic supplementary material:**

The online version of this article doi: (10.1186/s12883-017-0947-6) contains supplementary material, which is available to authorized users.

## Background

Cerebral watershed infarction (WSI), ischemic lesions between two non-anastomosing main arterial territories, can be classified as either cortical watershed infarction (CWI; or external watershed infarction) or internal watershed infarction (IWI; or subcortical watershed infarction), both of which can be further divided into subtypes. The mechanism of WSI has not been fully understood. Traditionally, hemodynamic impairment (HDI) has been widely accepted as a cause of WSI [1], but microemboli may also contribute to it [2].

It is unclear whether the mechanism differs among different types and subtypes of WSI since most previous studies either combined CWI and IWI into a single group or focused only on one type or subtype of WSI. Only a few studies [3–5] tried to find the differences in the characteristics of different WSI types but yielded controversial results. Therefore, whether the pathogenesis differs among them is unclear. In addition, cases where both CWI and IWI are present, which are named mixed-type infarction in our study, have never been included in previous studies.

In this study, we tried to distinguish the mechanisms of different types and subtypes of WSI. Based on the analysis of pre-existing results, we postulated that IWI was more relevant to HDI than CWI. Clinical courses and short-term clinical outcomes were also compared among different types of WSI.

## Methods

### Subjects

Patients with acute ischemic stroke admitted to the neurology department of Beijing Chao-Yang Hospital, Capital Medical University from August 2013 to January 2016 were identified. Only patients who met the following criteria were recruited: (1) admitted to the hospital within 7 days of symptom onset; (2) identified as WSI based on diffusion-weighted imaging(DWI) sequence; (3) completed evaluations including medical history, risk factors, cardiologic test, routine blood tests, stroke scales and cerebrovascular examinations. Potential sources of cardioembolism (PSCE) were identified if any of the following existed: recent myocardial infarction (<3 weeks), atrial fibrillation, dilated cardiomyopathy, acute bacterial endocarditis, mitral stenosis, prosthetic valve replacement, sick sinus syndrome or patent foramen [6].

### Ethics

Informed consent was obtained from patients for magnetic resonance imaging (MRI) and computed tomographic angiogram (CTA), and to the use of data for research. All medical procedures were performed only for clinical reasons. The study was approved by the Ethics Committee of Beijing Chao-Yang Hospital, Capital Medical University and was in accordance with the declaration of Helsinki.

### Imaging protocol

MRI was performed on the same 3.0 T Siemens scanner (Erlangen, Germany). The parameters of MR examination were as follows: axial T2-weighted (repetition time, 4500 ms; echo time, 93 ms), axial T1-weighted imaging (repetition time, 2000 ms; echo time, 9.2 ms), axial diffusion-weighted imaging (repetition time, 3300 ms; echo time, 91 ms), and coronal fluid-attenuated inversion recovery sequences (repetition time, 8000 ms; echo time, 86 ms).

CTA was performed on a dual-source CT scanner (Somatom Definition; Siemens Medical Solutions, Forchheim, Germany). For head and neck CTA, 70–80 ml non-ionic contrast agent (350 mg iodine/ml ioversol, Optiray 350, Mallinckrodt Pharmaceuticals) was injected into the antecubital vein at 5 ml/s followed by a 40-ml saline flush also at 5 ml/s. The CTA covered the area from just below the aortic arch to the vertex. CTA was performed in a helical scan mode using the following parameters: 64 × 0.6 collimation, 0.65 pitch, 120 kV, automatic exposure control with standard deviation of 10 and exposure range 100–700 mA, 0.625 mm and 3.0 mm slice thickness, 0.33 s rotation time, reconstruction filter FC43 and standard AIDR3D. The bolus tracker was set at an absolute threshold of 180 HU at the level of the descending aorta in dual energy mode.

### MRI imaging analysis

**Fig. 1:**
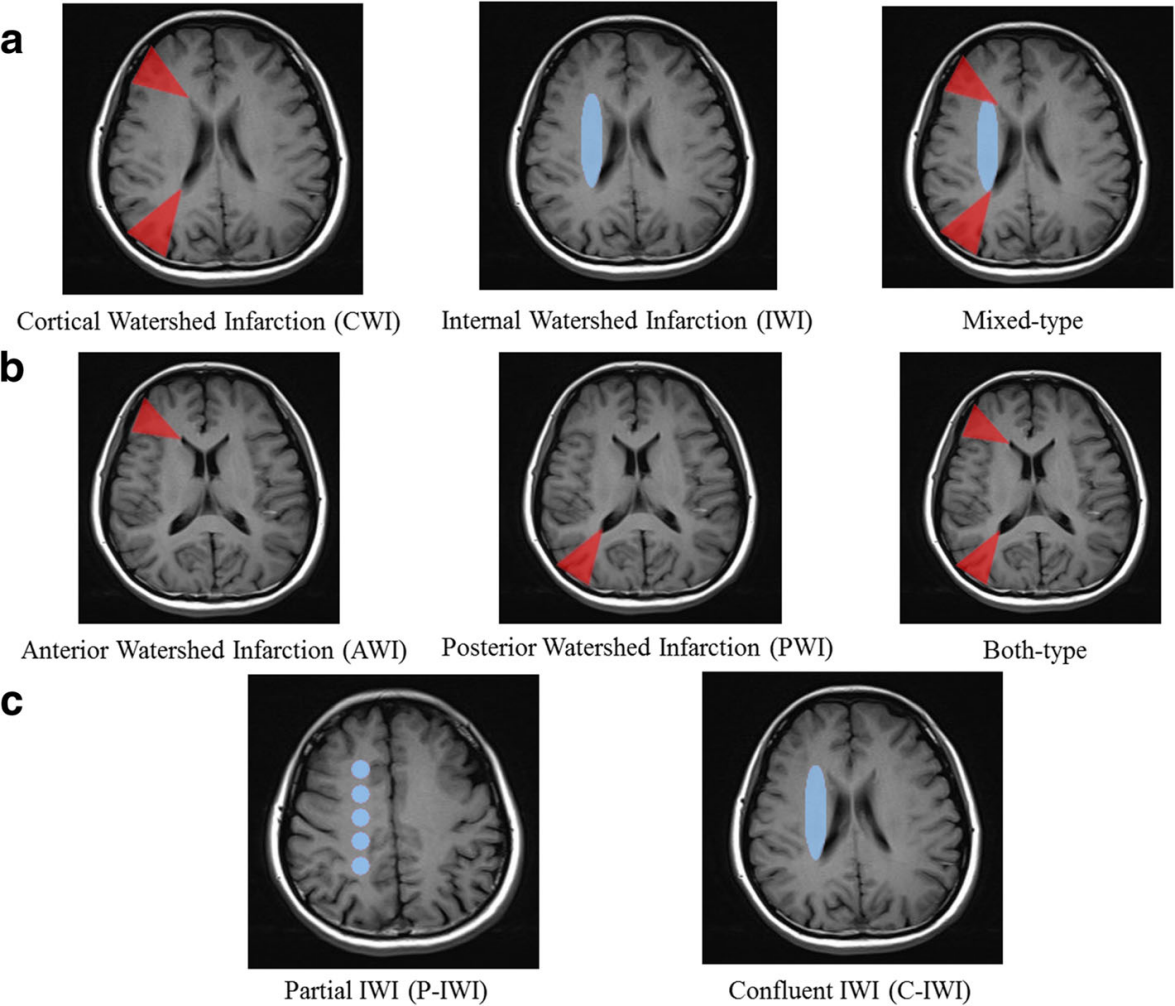
Classification of watershed infarction (WSI): (**a**) types of WSI; (**b**) subtypes of cortical watershed infarction (CWI); (**c**) subtypes of internal watershed infarction (IWI)

Two experienced neurologists (Lei Yang and Man Li) who were unaware of the patient’s clinical information typed the classification with templates [7]. CWI was defined as hyperintense areas on DWI sequence in the junctions of the anterior cerebral artery (ACA), middle cerebral artery (MCA), and posterior cerebral artery (PCA) territories, as a thin fronto-parasagittal wedge. CWI was further divided into anterior watershed infarction (AWI, between ACA and MCA), posterior watershed infarction (PWI, between MCA and PCA) and both-type infarction (AWI plus PWI). IWI was defined as hyperintense areas on DWI sequence between the deep and superficial perforating arteries of the MCA, and further divided into partial IWI (P-IWI, a single lesion or a chain-like, the so-called “rosary-like” pattern in the centrum semiovale) and confluent IWI (C-IWI, large cigar-shaped infarction alongside the lateral ventricle). A concurrence of CWI and IWI was identified as mixed-type infarction. Classifications of WSI, CWI and IWI is presented in Fig. 1.

We used North American Symptomatic Carotid Endarterectomy Trial (NASCET) method to measure the degree of cerebrovascular steno-occlusion as [8]: non-significant (< 50%), moderate (50% to 74%), severe (75% to 99%), or occluded. The critical stenosis was defined as >75% stenosis or occlusion. The κ-coefficient for interobserver agreement was 0.824 for the classification of WSI and 0.797 for the degree of cerebrovascular steno-occlusion.

### Demographic and clinical assessment

The following clinical information was collected: sex, age, history of hypertension (defined as treatment with antihypertensive medications or BP > 140/90 mmHg measured on several separate occasions), diabetes mellitus (defined as treatment with antidiabetic medications or fasting plasma glucose >7.0 mmol/L), hyperlipidemia (based on history), smoking (continuously smoking >1 cigarette a day for 6 months), stroke or transient ischemic attack (TIA) and coronary artery disease (CAD, defined as a history of myocardial infarction or angina pectoris), blood pressure (BP, recorded at admission) and results of laboratory tests including level of total cholesterol (TC), triglyceride (TG), high density lipoprotein (HDL), low density lipoprotein (LDL), glycosylated hemoglobin (HbA1c), albumin, plasma fibrinogen (Fib), homocysteine (HCY), high-sensitivity C-reactive protein (hs-CRP), serum creatinine (Cr) and uric acid (UA).

All patients received standard treatment in the stroke unit. The National Institutes of Health Stroke Scale (NIHSS) score was checked at day 1 and 7 after admission. The clinical course was defined by change of NIHSS score between the first and seventh day after admission as: 1) improved (NIHSS score decreased by ≥2 points); 2) stable (NIHSS score decreased by <2 points); and 3) deteriorated (NIHSS score increased after admission). Short-term clinical outcome was assessed by Modified Rankin Scale (mRS) 3 months after stroke onset via telephone follow-up and we defined poor outcome as mRS score > 3 points.

### Statistical analysis

All statistics were presented as mean ± standard deviation (SD) for continuous variables with normal distribution, median and interquartile range for continuous variables with non-normal distribution, and counts and proportions for categorical variables. Differences among the groups and subgroups were examined using Pearson χ^2^ test, one-way analysis of variance (ANOVA), or non-parametric test, depending on the nature of the variables being compared. The Bonferroni correction was used for post hoc comparisons to obtain an adjusted significance level for each domain-specific test: ≈0.05/3 = 0.017. Statistical significance was established at *P* < 0.05. Logistic regression analysis was applied to identify independent predictors of poor clinical outcome. Independent variables were age, sex, vascular risk factors, PSCE, clinical course, NIHSS score at admission, type of WSI, and the critical stenosis of ICA and MCA. The results were presented as estimates of relative risk by odds ratio (OR) with a 95% CI. Analysis was performed with Statistical Package for Social Sciences (SPSS version 24).

## Results

### Participants characteristics

During the study, 351 WSI patients were identified, among whom 11 with incomplete examinations were excluded. As a result, 340 patients were recruited with 220 (64.70%) males. Among the 340 WSI patients, 92 (27.10%) had CWI, 112 (32.90%) had IWI and 136 (40.00%) had mixed-type infarction. Details of clinical and demographic features of the patients in each group are presented in Table 1. The age, sex, risk factors and laboratory data did not differ among three groups.

**Table 1:** Clinical and demographic features of patients with different types of WSI

Variable	CWI (*n* = 92)	IWI (*n* = 112)	Mixed-type (*n* = 136)	*P*
Male, *n* (%)	54 (58.7)	73 (65.2)	93 (68.4)	0.321
Age, years	65.46 ± 10.42	66.12 ± 10.89	64.54 ± 11.26	0.429
Risk factors, *n* (%)				
Hypertension	60 (65.2)	79 (70.5)	93 (68.4)	0.718
Diabetes mellitus	27 (29.3)	36 (32.1)	43 (31.6)	0.903
Hyperlipidemia	48 (52.2)	60 (53.6)	63 (46.3)	0.480
PSCE	22 (23.9)	14 (12.5)	20 (14.7)	0.071
Smoking	53 (57.6)	72 (64.3)	82 (60.3)	0.613
Stroke / TIA	28 (30.4)	38 (33.9)	52 (38.2)	0.468
CAD	21 (22.8)	15 (13.4)	16 (11.8)	0.059
SBP, mmHg	147.43 ± 25.13	154.45 ± 23.07	152.87 ± 23.78	0.791
DBP, mmHg	85.32 ± 11.45	87.23 ± 12.87	86.24 ± 11.89	0.613
Laboratory data				
TC, mmol/L	4.32 ± 1.12	4.69 ± 0.93	4.54 ± 1.18	0.512
TG, mmol/L	1.23 (0.92, 1.89)	1.49 (0.98, 2.16)	1.37 (1.02, 2.09)	0.897
HDL, mmol/L	1.19 ± 0.31	1.36 ± 0.27	1.37 ± 0.39	0.318
LDL, mmol/L	2.52 ± 0.71	2.69 ± 0.67	2.62 ± 0.62	0.634
HbA1c, %	6.40 (5.60, 7.90)	6.50 (5.80, 8.10)	6.40 (5.70, 7.80)	0.692
Albumin, g/L	37.62 ± 4.87	37.66 ± 4.14	37.21 ± 4.54	0.941
Fibrinogen, mg/dl	334.76 ± 65.87	326.86 ± 71.23	313.23 ± 70.65	0.327
HCY, mmol/L	17.00 (14.00, 20.00)	19.00 (15.00, 24.00)	18.00 (15.00, 23.00)	0.093
Hs-CRP, mg/L	2.45 (1.17, 7.83) (1.06,8.65)	2.54 (1.21, 6.87)	2.64 (1.15, 5.45)	0.432
Cr, μmol/L	84.76 (70.87, 105.92)	85.16 (75.32, 97.98)	84.94 (73.66, 99.72)	0.675
Uric acid, μmol/L	321.87 ± 91.76	319.76 ± 98.65	312.55 ± 97.60	0.821

Data are presented as mean ± standard deviation, median (interquartile range) or counts (%)

*WSI* Watershed infarction, *CWI* Cortical watershed infarction, *IWI* Internal watershed infarction, *N* Number of persons, *PSCE* Potential sources of cardioembolism, *TIA* Transient ischemic attack, *CAD* Coronary artery disease, *SBP* Systolic blood pressure, *DBP* Diastolic blood pressure, *TC* Total cholesterol, *TG* Triglyceride, *HDL* High density lipoprotein, *LDL* Low density lipoprotein, *HbA1c* Glycosylated hemoglobin, *HCY* Homocysteine, *Hs-CRP* High-sensitivity C-reactive protein, *Cr* Serum creatinine

### Incidence of critical cerebrovascular stenosis in different WSI types and subtypes

**Fig. 2:**
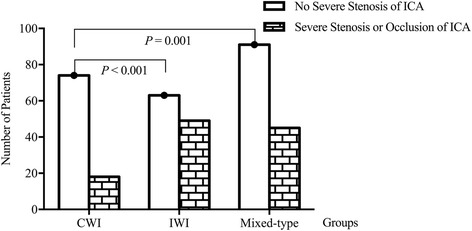
Comparison of the incidence of critical internal carotid artery (ICA) stenosis among different types of WSI. The incidence of critical stenosis of ICA differed among three groups of WSI (*P* = 0.001), more patients in the IWI group were found to have critical ICA stenosis than those in the CWI group (43.80% versus 19.60%; *P* < 0.001)

The incidence of critical stenosis of internal carotid artery (ICA) differed among three groups of WSI (*P* = 0.001), but that of other cerebral vessels did not. In further comparison, more patients in the IWI group were found to have critical ICA stenosis than those in the CWI group (43.80% vs. 19.60%; *P* < 0.001, Fig. 2). These results are presented in Additional file 1: Table S1.

**Fig. 3:**
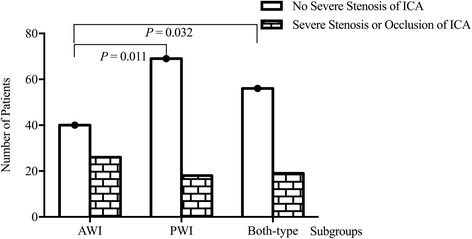
Comparison of the incidence of critical ICA stenosis among different subtypes of CWI. The incidence of critical stenosis of ICA differed among three subgroups of CWI (*P* = 0.032), patients with AWI were more prone to critical ICA stenosis than those with PWI (39.40% versus 20.70%; *P* = 0.011)

**Fig. 4:**
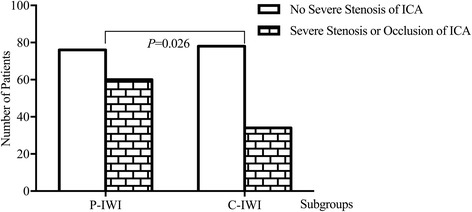
Comparison of the incidence of critical ICA stenosis between different subtypes of IWI. Critical ICA stenosis was more common in the P-IWI subgroup than in the C-IWI subgroup (44.10% versus 30.40%; *P* = 0.026)

Among 228 patients who had CWI (including 136 mixed-type patients), patients with AWI were more prone to critical ICA stenosis than those with PWI (39.40% vs. 20.70%; *P* = 0.011, Fig. 3). Among 248 patients who had IWI (including 136 mixed-type patients), critical ICA stenosis was more common in the P-IWI subgroup than in the C-IWI subgroup (44.10% vs. 30.40%; *P* = 0.026, Fig. 4).

### Differences in clinical course of hospitalization and prognosis at 90th day

Although NIHSS scores at admission did not differ significantly among the groups, clinical course during the first week after admission was significantly different: only 13.0% of CWI patients deteriorated compared with 33.9% of IWI patients and 25.7% of patients who had mixed-type infarction. Further analysis showed that patients with IWI were more prone to deterioration than those with CWI (*P* = 0.003). Follow-up prognosis 3 months after stroke showed significantly more IWI patients with a poor outcome than CWI patients (*P* = 0.014). Details of the clinical course and prognosis of the 3 groups are presented in Table 2.

**Table 2:** Clinical course and prognosis at 90th day of patients with different types of WSI

Variable	CWI (*n* = 92)	IWI (*n* = 112)	Mixed type (*n* = 136)	*P*
Initial NIHSS Score	3 (1, 5)	3 (1, 6)	3 (1, 5)	0.423
Clinical course, *n* (%)				
Improving	33 (35.9)	18 (16.1)	26 (19.1)	0.002
Stable	50 (54.3)	56 (50.0)	76 (55.9)	0.641
Deteriorated	12 (13.0) ^a^	38 (33.9) ^a^	35 (25.7)	0.003
Prognosis at 90th day				
Poor outcome, *n* (%)	21 (22.8) ^a^	47 (42.0) ^a^	50 (36.8)	0.014

Data are presented as counts (%)

*WSI* Watershed infarction, *CWI* Cortical watershed infarction, *IWI* Internal watershed infarction, *NIHSS* The National Institutes of Health Stroke Scale, *N* number of persons

^a^ Significant difference between CWI and IWI group

After adjustment for confounding factors, multivariate logistic regression analysis revealed that the presence of IWI (OR, 5.231; 95% CI, 1.687 to 9.703; *P* = 0.008), critical stenosis of ICA (OR, 2.284; 95% CI, 1.376 to 5.662; *P* = 0.012), and the initial NIHSS score (OR, 1.877; 95% CI, 1.176 to 2.012; *P* = 0.004) were independently associated with poor outcome 3 months after stroke.

## Discussion

In this study, we demonstrated that IWI, especially the P-IWI subtype, is associated with HDI and that IWI patients are more prone to poor prognosis. To our knowledge, this is the first study to investigate the mechanisms of different types and subtypes of WSI, for particular, the mixed-type infarction defined here as the concurrence of CWI and IWI.

Traditionally, WSI is mainly considered as a result of critical stenosis of main arteries, generally with precipitating factors such as acute hypotensive events, cardiopulmonary bypass and anesthesia. Another widely accepted hypothesis is that microemboli from the heart or artery-to-artery preferentially propagated to watershed areas because of their diameters [9].

Then, Caplan LR et al. [10] proposed a synergetic interaction theory: reduced perfusion weakens the ability to wash out microemboli, particularly within the border-zone areas. But another study [2] found that less microembolic signals (MES) were detected in patients with severe ICA stenosis and more in those with a lower degree of ICA stenosis. To explore the role of HDI and microemboli in the pathogenesis of WSI, Moustafa RR et al. [11] applied positron emission tomography and transcranial doppler in TIA/ minor stroke patients whose carotid stenosis was ≥50%, and finally found that there was no significant difference between these two factors and no evidence of synergistic effect.

However, despite their inspirations, previous studies are limited in the following aspects. Firstly, CWI and IWI have been combined in most studies into a single group as a result of limited clinical cases. Some reports focused on either only CWI [12, 13] or IWI [14–16], which precluded the detection of different pathogeneses for different types and subtypes of WSI. Moreover, chronic cerebral infarction was not ruled out in many previous studies due to the absence of DWI sequence. Furthermore, the concurrence of both CWI and IWI, which is defined as mixed-type in our study, has not been included in any previous studies to our knowledge.

So far, only five studies [3, 5, 17–19] have explicitly examined the pathophysiological differences of CWI and IWI, but three of them did not use DWI sequence and two of them had selection bias for enrolling patients with cerebrovascular stenosis or occlusion.

The results of our study demonstrated an association between IWI and critical stenosis of ICA, whereas the association is weakest in CWI patients. Consistent with previous research [14], these findings supported the theory that HDI may be the main cause of IWI. The relationship between CWI and HDI appears more complicated with previous research stating that [18] artery-to-artery embolism might play an important role in isolated CWI.

The susceptibility of internal border-zone area to HDI is probably due to low perfusion pressure in perforating medullary arteries, the most distal branches of the ICA with insufficient collateral supply of the deep perforating lenticulostriate arties.

To the best of our knowledge, no existing research has directly compared the hemodynamics of CWI subtypes. The available data [20] suggest that HDI is more frequently documented in the anterior border-zone area. Furthermore, for CWI concurrent with IWI, the AWI subtype had a higher incidence than the PWI subtype [21]. We found that AWI was prone to association with the critical stenosis of ICA. The vulnerability of the anterior border-zone area may be attributed to the fact that MCA and ACA are supplied only by ICA; thus stenosis or occlusion of ICA will cause AWI, and inefficient collateralization will further worsen the perfusion. Conversely, severe stenosis or occlusion of the vertebro-basilar system, or a fetal-type PCA may be responsible for PWI, which is probably rarer than the former.

A previous study [18] revealed that the rosary-like infarction in the centrum semiovale, which was identified as P-IWI in our study, appeared to be associated with HDI. But Moustafa RR et al. [22] found that in addition to HDI, microemboli might also play a role in the pathogenesis of rosary-like infarction. But few studies have compared the difference between C-IWI and P-IWI precisely. We found that critical ICA stenosis was more common in P-IWI patients than in C-IWI patients. This was contrary to a previous study [16] which identified 14 C-IWI and 13 P-IWI and found that occlusive diseases of ICA were more prevalent in C-IWI, but it was highly likely to be limited by the small sample size. Internal border-zone area is located in the junction of the ACA and MCA superficial perforators, a special anatomical location that shapes its sensitivity to hypoperfusion. Thus, the centrum semiovale, which represents the most distal region perfused by the ICA, is most vulnerable to HDI.

We demonstrated that patients with IWI had worse hospital courses and poor prognosis 3 months post stroke, and that IWI was an independent risk factor for poor outcome. A previous study [15] also identified IWI as a predictor of early neurological deterioration in minor stroke patients. These results suggest that more intensive care is necessary for patients with IWI, especially P-IWI, so as to improve perfusion.

Our study is also limited for the following reasons. Firstly, the number of patients in each subtype is relatively small although our study is already the largest in scale to date. Secondly, the location of border-zone is sometimes hard to define because vascular territories may vary greatly among individuals. Thirdly, our study was undertaken in the Chinese population in whom MCA stenosis is more prevalent, but there was no significant difference of the incidence of significant MCA stenosis or occlusion among 3 types of WSI, which need to be further studied.

## Conclusion

IWI, the P-IWI subtype in particular, seems to be related to HDI and poor prognosis, whereas CWI shows a weaker correlation with ICA steno-occlusion. Better understanding of the different pathogenesis of WSI would help identify patients at risk and prevent clinical deterioration.

## Additional file

Additional file 1: Table S1. Incidence of critical cerebrovascular stenosis in different WSI types. (DOCX 17 kb)

## Abbreviations

ACA: Anterior cerebral artery
ANOVA: One-way analysis of variance
AWI: Anterior watershed infarction
BP: Blood pressure
CAD: Coronary artery disease
C-IWI: Confluent IWI
Cr: Creatinine
CTA: Computed tomographic angiogram
CWI: Cortical watershed infarction
DBP: Diastolic blood pressure
DWI: Diffusion-weighted imaging
Fib: Fibrinogen
HbA1c: Glycosylated hemoglobin
HCY: Homocysteine
HDI: Hemodynamic impairment
HDL: High density lipoprotein
hs-CRP: High-sensitivity C-reactive protein
ICA: Internal carotid artery
IWI: Internal watershed infarction
LDL: Low density lipoprotein
MCA: Middle cerebral artery
MRI: Magnetic resonance imaging
mRS: Modified Rankin Scale
NIHSS: The National Institutes of Health Stroke Scale
OR: odds ratio
PCA: Posterior cerebral artery
P-IWI: Partial IWI
PWI: Posterior watershed infarction
SBP: Systolic blood pressure
SD: Standard deviation
TC: total cholesterol
TG: Triglyceride
TIA: Transient ischemic attack
UA: Uric acid
WSI: Watershed infarction
