# Supplementary material for: Clinical features and the degree of cerebrovascular stenosis in different types and subtypes of cerebral watershed infarction
Source: BMC Neurol. 2017 Aug 29;17:166. doi: 10.1186/s12883-017-0947-6 (PMC5576255; doi:10.1186/s12883-017-0947-6)
Supplement: Additional file 1: Table S1. — Incidence of critical cerebrovascular stenosis in different WSI types. (DOCX 17 kb) [file 12883_2017_947_MOESM1_ESM.docx]

Table S1 Incidence of critical cerebrovascular stenosis in different WSI types

|  | CWI  (n = 92) | IWI  (n = 112) | Mixed-type  (n = 136) | *P* |
| --- | --- | --- | --- | --- |
| Internal Carotid Artery | 18 (19.60) † | 49 (43.80) † | 45 (33.10) | 0.001 |
| Middle Cerebral Artery | 36 (39.10) | 59 (52.70) | 71 (52.20) | 0.093 |
| Anterior Cerebral Artery | 10 (10.90) | 19 (17.00) | 14 (10.30) | 0.242 |
| Posterior Cerebral Artery | 14 (15.20) | 17 (15.20) | 13 (9.60) | 0.316 |
| Vertebral Artery | 15 (16.30) | 28 (25.00) | 26 (19.10) | 0.279 |
| Basilar Artery | 11 (12.00) | 14 (12.50) | 17 (12.50) | 0.991 |
| Subclavian Artery | 9 (9.80) | 12 (10.70) | 16 (11.80) | 0.893 |
| Common Carotid Artery | 6 (6.50) | 13 (11.60) | 12 (8.80) | 0.449 |
| External Carotid Artery | 4 (4.30) | 3 (2.70) | 4 (2.90) | 0.774 |

Data are presented as counts (%).

*WSI* Watershed infarction, *CWI* Cortical watershed infarction, *IWI* Internal watershed infarction, *N* number of persons; † Significant difference between CWI and IWI group.
